# Protective Effects of Total Glycoside From *Rehmannia glutinosa* Leaves on Diabetic Nephropathy Rats via Regulating the Metabolic Profiling and Modulating the TGF-β1 and Wnt/β-Catenin Signaling Pathway

**DOI:** 10.3389/fphar.2018.01012

**Published:** 2018-09-11

**Authors:** Xinxin Dai, Shulan Su, Hongdie Cai, Dandan Wei, Hui Yan, Tianyao Zheng, Zhenhua Zhu, Er-xin Shang, Sheng Guo, Dawei Qian, Jin-ao Duan

**Affiliations:** Jiangsu Collaborative Innovation Center of Chinese Medicinal Resources Industrialization, State Key Laboratory Cultivation Base for TCM Quality and Efficacy, Nanjing University of Chinese Medicine, Nanjing, China

**Keywords:** total glycoside extracted from leaves of *Rehmannia*, Dihuangye total glycoside capsule, metabolomics, diabetic nephropathy, TGF-β1, Wnt/β-catenin signaling pathway

## Abstract

*Rehmannia glutinosa* Libosch (RG), is officially listed in the *Chinese Pharmacopoeia* and is widely used in China. The leaves of RG (LR) is an important vegetative organ of the plant. At present, the total glycosides of RG (TLR) were extracted from RG, and developed a national second class of new drugs to the Dihuangye total glycoside capsule (DTG). Additionally, DTG has the effect of nourishing yin and tonifying kidney, promoting blood circulation and blood cooling, and applicable to chronic glomerulonephritis mild to Qi and Yin Deficiency. Moreover, diabetic nephropathy (DN) rats model was induced by intraperitoneal injection of a small dose of streptozotocin (45 mg/kg) and high-fat diet and plus 5% glucose drinking water. Over 15 days, after oral administration TLR and DTG in DN rats, samples from serum, urine and kidney were collected for biochemical indicators measurements, pathological analysis, western blotting and metabolomics. Therefore, the analytical results of biochemical indicators, histopathological observations and western blotting showed that TLR and DTG exhibited a significant effect in renal protection. And 27 endogenous metabolites (12 in serum and 15 in urine) could be tentatively identified in the process of DN in rats using metabolomics method. Those endogenous metabolites were chiefly involved in sphingolipid metabolism; pentose, glucuronate interconversion; terpenoid backbone biosynthesis; purine metabolism and retinol metabolism. After drug intervention, these endogenous metabolites turned back to normal level some extent (*P* < 0.05). Furthermore, TLR and DTG prevent high glucose-induced glomerular mesangial cells (GMCs) by inhibiting TGF-β1 and Wnt/β-catenin signaling pathway, providing a powerful supports to develop a new therapeutic agent for DN. This study paved the way for further exploration of the pathogenesis of DN, early diagnosis and the evaluation of curative effect.

## Introduction

Traditional Chinese medicine (TCM) with multi-components and multi-targets has been used in clinical for over 4000 years in China due to its efficacy and low side-effects (Yokozawa et al., 1986). RG, belonging to Scrophulariaceae family, is widely distributed in China, and its root tubers are commonly called “Dihuang" in China. So far large quantity of iridoids and phenylethanoid glycosides have been isolated from the root and leaves of RG, together with other types of active principles. “Dihuang" has the significant effects on cardiovascular diseases (Liu et al., 2011), neuroprotection (Cai et al., 2011), diabetes and its complications (Zhang et al., 2014), osteoporosis (Zhang Z. et al., 2013), and hyperlipidemia (Zhang R. et al., 2013). Currently, with the deep exploration of RG, the leaves of RG (LR) is gaining increased attention around the world. In order to find the possibility to be used as medicine, people are beginning to pay attention to the development and utilization of LR. At present, LR is officially listed in the *Beijing standard of Chinese herbal medicines*, and it has the effect of heat-clearing, promoting blood circulation, supplementing Qi and nourishing yin and tonifying kidney (Beijing Municipal Health Bureau, 1988). The total glycosides of RG (TLR) were extracted from RG. The major active components of DTG is the phenylethanoid glycosides, which is extracted from LR, and phenylethanoid glycosides are a class of natural glycosides containing hydroxyl groups, methoxy substituted phenethyl or cinnamoyl groups, usually containing β-glucose as the parent nucleus, and are widely present in dicotyledonous plants (Jiménez and Riguera, 1994). DTG can increased glomerular permeability and reduced glomerular hyperfiltration, thus reducing proteinuria and the protection of renal function (Shen et al., 2010).

Diabetic nephropathy (DN) is one of the most common microangiopathy in diabetic patients, and renal fibrosis is the main cause of death in patients with DN (Lv et al., 2015). At present, the clinical diagnosis of DN mainly depending on the urinary albumin excretion rate detection, however, urinary microalbumin content may be affected by many factors such as obesity, insulin resistance and there are limitations (Weir, 2007). Therefore, it is necessary to find an early diagnostic marker with high sensitivity and specificity. To the best of our knowledge, there is no unified theory about the pathogenesis of DN. The current research focuses on the aspects of glucose metabolism, lipid metabolism, oxidative stress (Oberg et al., 2004; Chen and Quilley, 2008; Balakumar et al., 2009), and so on. Studies have shown that Wnt/β-catenin signaling plays a prominent role in cell differentiation, adhesion, survival, and apoptosis and is involved in glomerular cell proliferation and renal fibrosis, which affected the occurrence and development of DN (Xiao et al., 2013).

Metabolomics, based on the dynamic changes of endogenous metabolites in organisms, revealing the overall physiological status in responding to pathophysiological stimuli or genetic, environmental, or lifestyle factors (Pereira and Chang, 2004), and it has been widely used in the diagnosis of diseases, marker screening and pathogenesis research as a kind of technical means to study the dynamic metabolism of biological organism or tissue cells in recent years (Odunsi et al., 2005; Hodavance et al., 2007; Hwang et al., 2010). Metabolomics is regarded as “biochemical phenotypes” of the whole functional state of organism, and can be real-time, sensitive and real to express the response and regulation of the overall functional state of the organism under various external factors (Nicholson and Lindon, 2008).

Therefore, this study parallel simultaneous analysis of DN rats and normal rats of serum and urine, markers of different metabolites, and to study the effect of DTG and TLR on DN by analyzing the regulation of different metabolites. Moreover, the high glucose-induced GMCs model *in vitro* evaluate the efficacy of DTG and TLR on DN by determination of TGF-β1, Wnt4 and β-catenin proteins expression levels.

## Materials and Methods

### Chemicals and Instruments

UPLC-grade acetonitrile was purchased from Merck (Darmstadt, Germany), formic acid and STZ were purchased from Sigma-Aldrich (Sigma, St. Louis, MO, United States). HPLC-grade acteoside was purchased from the National Institutes for Food and Drug Control (Beijing, China) and the purity is above 98%. BUN reagent kit, LDL reagent kit, T-CHO reagent kit, TG reagent kit, Scr reagent kit, UP reagent kit, serum β2-microglobulin (β2-MG) reagent kit were bought from Nanjing Jiancheng Bioengineering Institute (Nanjing, China). Huangkui capsule was purchased from SZYY Group Pharmaceutical Limited; Irbesartan was purchased from Shenzhen Haibin Pharmaceutical Co., Ltd. DMEM and F12 were purchased from GIBCO, America; TGF-β1, Wnt4 and β-catenin first antibody were purchased from Abcam (Cambridge, United Kingdom). All other chemicals and reagents used in this study were of analytical grade and made in China.

Waters Acquity^TM^ Ultra Performance LC system (Waters, United States) equipped with a Quattro Micro MS spectrometer and a Waters Xevo TM G2 QTof MS (Waters MS Technologies, Manchester, NH, United States). Deionized water was purified on a Milli-Q system (Millipore, Bedford, MA, United States). Mass Lynx v4.1 workstation was adopted to analyze the data, and Ultra-high speed centrifuge at low temperature (Thermo Scientific, United Kingdom); DMI3000M microscope (Leica, Germany) were used.

### Preparation of TLR and DTG

The plant material, LR were purchased from Henan farmers market, and identified by the Prof. Jin-Ao Duan (Department of Nanjing University of Chinese Medicine). Fresh LR were vacuum-dried in 80°C and ground into powder. The vacuum-dried LR (500 g) was extracted with 6 L 80% alcohol three times and 2 h each time with reflux extraction. In rotary evaporation instrument reduced pressure concentration, as TLR low dose oral solution. Concentrated on dose in the twice as high dose oral solution.

**FIGURE 1:**
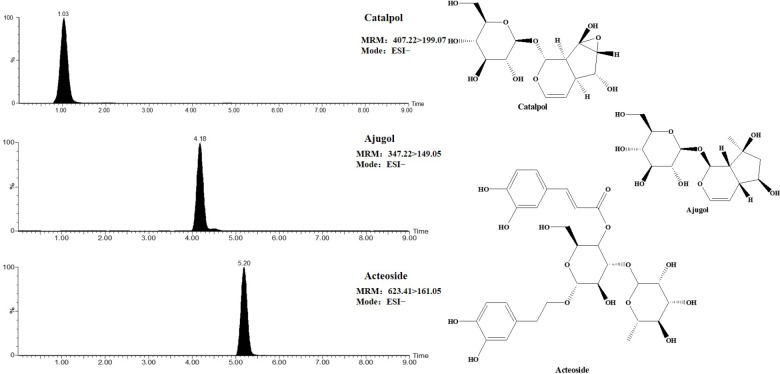
MRM chromatogram and structure of catalpol, ajugol, and acteoside.

Before the experiment, the chemical of TLR and DTG were previously established by the UPLC-TQ-MS. And the main components of TLR identified mainly as catalpol, ajugol, and acteoside, the contents of above components were 0.6326%, 0.4105%, and 0.6833%, respectively. DTG were bought from Sichuan Meidakang Pharmaceutical Co., Ltd., the main component is acteoside, and the content of the acteoside in the DTG was 13.61%. The MRM chromatogram and structure of catalpol, ajugol and acteoside were presented in **Figure 1**. Moreover, we made the content of acteoside in TLR oral solution and DTG oral solution is consistent for rats.

### Experimental Animals

All experiments were conducted with male Sprague–Dawley (SD) rats weighing 200–240 g purchased from Experimental animal center of Zhejiang Province (Zhejiang, China; Certificate no. SCXK 2014-0001). Animals were housed in cages with a constant humidity (ca. 60% ± 2%) and temperature (ca. 23 ± 2°C) and with a light/dark cycle of 12 h. The animals were underwent an adaptation period of 7 days, during which they were allowed unlimited access to chow and tap water.

After the 7-day acclimation period, animals were randomly divided into eight groups with 6 in each group: control group (normal saline, C), model group (normal saline, M), Huangkui capsule group (0.75 g/kg⋅d, HK), Irbesartan group (27 mg/kg⋅d, YX), TLR low dose group (4.3 g/kg⋅d, DHYL), TLR high dose group (7.2 g/kg⋅d, DHYH), DTG low dose group (216 mg/kg⋅d, JNL), DTG high dose group (360 mg/kg⋅d, JNH). Expect for control group rats, the rats in other groups were fed with high-fat diet (59% basic mice feed, 20% sugar, 18% lard, and 3% egg yolk) and 5% glucose water for 21 days, then, the rats were injected intraperitoneally with STZ (45 mg/kg) dissolved in 0.1 mol/L sodium citrate buffer (pH 4.5) to induce DN model. 72 h after the injection of STZ, rats with FBG levels of 16.7 mmol/L and above were considered to be in diabetic state and were used for the study. Moreover, in this study, Huangkui capsule and Irbesartan were selected as positive control drug. The animal dose of Huangkui capsule and Irbesartan were extrapolated from the human daily dose, using the body surface area normalization method. The formula for dose translation was as follows: human clinical equivalent dose of medicine × 70 × 0.018/0.2. The dose of JNL and JNH were three times and five times of the clinical equivalent dose, respectively. Moreover, TLR are about 20 times the corresponding dose of DTG. Then, the rats were given corresponding drug once a day by gastric gavage for 15 days, and the control group rats were given the same volume of saline at the same time. This study was carried out in accordance with the recommendations of the Institutional Animal Ethics Committee of Nanjing University of Chinese Medicine (Nanjing, China). The protocol was approved by the Institutional Animal Ethics Committee of Nanjing University of Chinese Medicine.

### Sample Collection

During the experimental period, body weights were recorded at 3-days intervals, FBG levels were measured by a One Touch Ultra II blood glucose monitoring system (Life Scan, Milpitas, CA, United States) by tail vein at 3-days intervals (at 8 a.m). Prior to the experiments, rats were put individually in the metabolism cages for 24-h urinary collection, all urine samples were immediately centrifugated at 3,000 rpm for 10 min after collection, and the supernatants were separated and stored at -80°C until analysis. At the end of study, rats were sacrificed under anesthesia by intraperitoneal injection of chloral hydrate (330 mg/kg body weight), and blood was collected for concentration of biochemical parameters and metabolomics study. Then, the blood samples were centrifugated at 3,000 rpm for 10 min, and the serum samples were separated and stored at -80°C until analysis. The right kidney was removed with one piece fixed with 10% neutral-buffered formalin for histologic examination. Renal cortex was isolated from the other piece and frozen in liquid nitrogen for western blotting analysis.

### Biochemical Indicators Measurements, Pathological Analysis and Western Blotting

The therapeutic efficacy of TLR and DTG on DN rats was evaluated for the levels of UP in urine and FBG, LDL, T-CHO, TG, Scr, BUN and β2-MG in serum, which were detected followed by the description supplied by kits manufacturer. Part of the renal cortex were performed for HE staining in order to observe pathological changes in renal tissue, degrees of fibrosis tissue hyperplasia, structures of glomeruli and tubules through electron microscope (200×), and another part of the renal tissues samples were analyzed by western blotting for detecting the expression levels of Wnt4, β-catenin and TGF-β1 protein.

### GMC Cells Culture

Glomerular mesangial cells were provided by Nanjing KeyGen Biotechnology Development Co., Ltd. The complete medium was low glucose DMEM with 10% FBS and 1% penicillin-streptomycin solution. The GMCs were cultured in 37°C, 5% CO_2_ and saturated humidity. The GMCs were plated on 96-well plates, pretreated with DMEM low glucose (5 mmol⋅L^-1^), incubated at 37°C and 5% CO_2_ for 6 h, and then were synchronized. The experimental groups were divided as follows: control group (C), which without intervention factor; model group (M), which cells were cultured in high glucose (30 mmol⋅L^-1^) DMEM solution; Acteoside (MRHTG) group (acteoside is stable in 0.1% DMSO in 24 h by analyzing its contents), TLR group (DHY) and DTG group (JN) at five dosages (5, 10, 25, 50, and 100 μmol⋅L^-1^), respectively. Cellular count was used to detect the number of cells in each group after incubation for 48 h, and then determined the total protein content of each group by BCA kit. The ratio of total cell protein/cell number was calculated and expressed as the total amount of protein (μg) contained in 10^3^ cells as an indicator of GMCs hypertrophy. Moreover, these cells were prepared for the western blotting to examine the expression levels of TGF-β1, Wnt4 and β-catenin protein.

### Sample Preparation and UPLC-QTOF/MS Analysis

All serum and urine samples were thawed at room temperature before preparation. The 300 μL acetonitrile was added to 100 μL serum samples to precipitated protein, and vigorously mixed for 60 s. The 100 μL acetonitrile was added to 100 μL urine samples to remove proteins and vigorously mixed for 30 s. All the samples were centrifuged at 13,000 rpm for 10 min at 4°C. Finally, the supernatant was injected to UPLC-QTOF/MS analysis.

Metabolites separation was performed using a Waters Acquity^TM^ Ultra Performance LC system (Waters, United States) equipped with a Waters Xevo^TM^ G2 Q/TOF-MS (Waters MS Technologies, Manchester, NH, United States). An aliquot of 2 μL of sample solution was injected on an Acquity UPLC BEH C_18_ (100 mm × 2.1 mm, 1.7 μm, Waters Corporation, Milford, CT, United States) at 35°C and the flowrate was 0.4 mL/min. The optimal mobile phase consisted of water (A) (containing 0.1% formic acid) and acetonitrile (B). The optimized UPLC elution conditions for serum were: 0 ∼ 3 min, 95% ∼ 55% A; 4 ∼ 13 min, 55% ∼ 5% A; 13 ∼ 14 min, 5% A. The optimized UPLC elution conditions for urine were: 0 ∼ 8 min, 95% ∼ 70% A; 8 ∼ 11 min, 70% ∼ 30% A; 11 ∼ 13 min, 30% ∼ 5% A; 13 ∼ 14 min, 5% A. The autosampler was maintained at 4°C.

Mass spectrometry was performed using a Xevo^TM^ G2 QTof (Waters MS Technologies, Manchester, NH, United States), a quadrupole and orthogonal acceleration time-of- flight tandem mass spectrometer. Leucine-enkephalin was used as the lock mass generating an [M+H]^+^ ion (*m/z* 556.2771) and [M-H]^-^ ion (*m/z* 554.2615) in positive and negative modes, respectively. The concentration of Leucine-enkephalin 200 pg/mL and the infusion flow rate was 100 μL/min to ensure accuracy during the MS analysis via a syringe pump. Data were collected in centroid mode from 100 to 1000 *m/z*. For both positive and negative electrospray modes, the capillary and cone voltage were set at 3.0 kV and 30 V, respectively. The desolvation gas was set to 600 L/h at a temperature of 350°C, the cone gas was set to 50 L/h and the source temperature was set to 120°C. The data acquisition rate was set to 30 ms, with a 0.02 s interscan delay.

In addition, 10 serum (or urine) samples were randomly selected from each group and mixed together as the quality control (QC) samples, respectively. The QC sample was used to optimize the condition of UPLC-QTOF/MS, as it contained most information of whole samples. The QC samples were injected six times at the beginning of the running in order to condition or equilibrate the system and then every ten samples to further monitor the stability of the analysis. Every day, after the instrument was calibrated, the QC sample was firstly analyzed to test the stability of the instrument in order to ensure consistent performance of the system.

### Metabolomics Data Processing and Analysis

All of the data acquisition and analyses of data were controlled by Waters MassLynx v4.1 software. The multivariate data matrix was analyzed by *EZ*info software 2.0 (Waters Corp., Milford, CT, United States). The main parameters include: retention time range 0 ∼ 14 min; mass ratio *m/z* 100 ∼ 1000, mass tolerance range 0.01 Da, peak intensity threshold 50, quality window 0.05 Da, retention time windows 0.20 min, automatic detection of 5% peak height and noise. The intensity of each ion was normalized with respect to the total ion count to generate a data matrix that consisted of the retention time, *m/z* value, and the normalized peak area.

The resultant data matrices were introduced to *E*Zinfo 2.0 software for principal component analysis (PCA), partial least-squares-discriminant analysis (PLS-DA) and orthogonal projection to latent structures (OPLS-DA) analysis. From the OPLS-DA, various metabolites could be identified as being responsible for the separation between control group and model group and were therefore viewed as potential markers. Potential markers of interest were extracted from S-plots constructed following analysis with OPLS-DA, and variables that had significant contributions to discrimination between groups were subjected to further identification of the molecular formula.

The variable importance (VIP) in the projection value is a weighted sum of squares of the PLS weights, and the variables with VIP >1 were considered to be influential for the separation of samples in the score plots generated from OPLS-DA analysis. In all experiments, confidence level was set at 95% to determine the significance of difference (*P* < 0.05). Those variables were eventually selected as potential biomarkers. The PLS-DA score plots were described by the cross-validation parameter *R*^2^*Y* and *Q*^2^, which represents the total explained variation for the *X* matrix and the predictability of the model, respectively. Excellent models are obtained when the cumulative values of *R*^2^*Y* and *Q*^2^ are above 0.8. The relative distances between administration groups and control group from PLS-DA score plot was calculated with the average value (*x*-axis and *y*-axis) of all samples of the control group as the referenced point (Li et al., 2010). This quantitative value was used as an indicator of pharmacodynamic evaluation of metabolomics, and it solves the problem of lack of accurate and quantitative evaluation methods for many pharmacodynamics.

### Metabolites Identification and Construction of Metabolic Pathway

Potential metabolites of DN selected were identified according to the determination of the accurate *m/z*, retention time, and typical MS/MS fragment and pattern of the potential biomarkers above, which were obtained in the positive and negative ion modes. The construction of metabolic pathway was performed with Metabo Analyst, which is a web-based tool for visualization of metabolomics^1^ based on database source including the KEGG^2^ and the HMDB^3^ databases for searching. The retention time and typical MS/MS fragment and pattern had great avail to narrow the range of possible molecules.

## Results

### Determination of Biochemical Indicators, Histopathological Observations and Western Blotting

**FIGURE 2:**
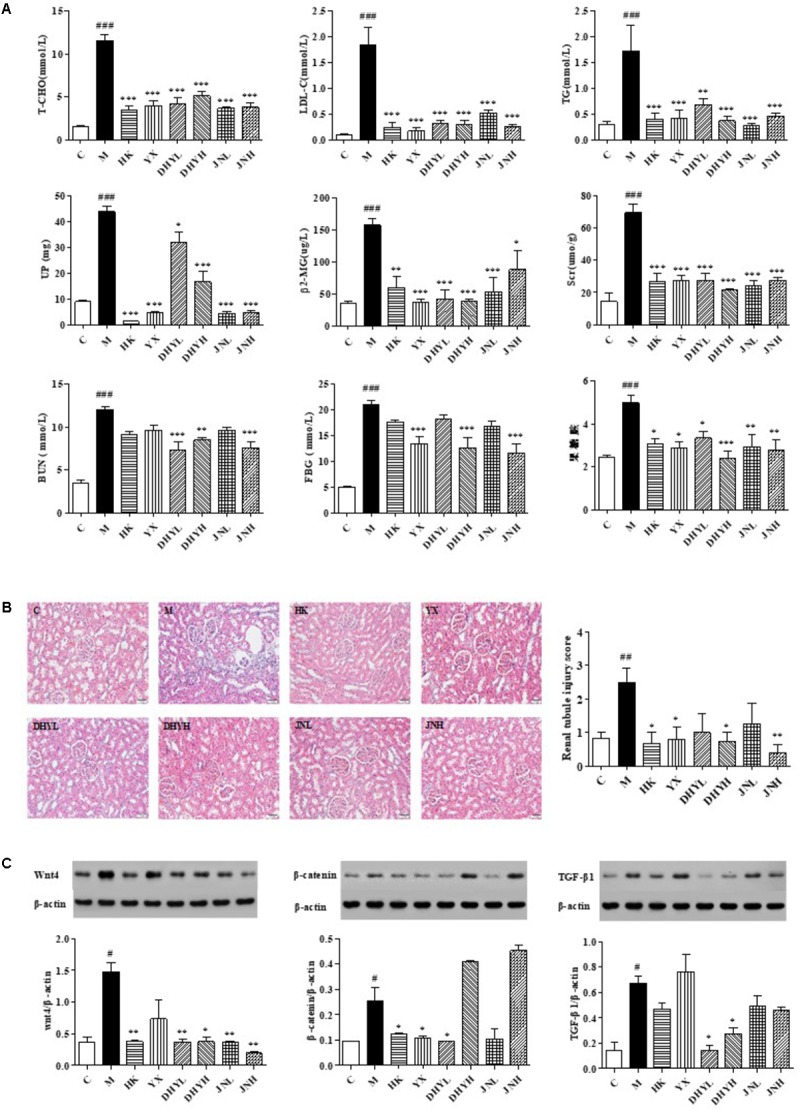
Determination of biochemical indicators and analysis of renal pathological section and Western blotting among control group, model group and administered group. **(A)** Determination of FBG, LDL, T-CHO, TG, Scr, β2-MG in serum, and BUN, UP in urine. **(B)** HE staining of kidney biopsy (200×). **(C)** Western blotting analysis of renal tissues of control group, HK group, YX group, DHYL group, DHYH group, JNL group and JNH group. ^#^*P* < 0.05, ^##^*P* < 0.01, ^###^*P* < 0.001: DN model group vs. control group; ^∗^*P* < 0.05, ^∗∗^*P* < 0.01, ^∗∗∗^*P* < 0.001: after treatment by HK, YX, DHYL, DHYH, JNL and JNH vs. DN model group. Data were presented as the mean ± SE.

The analytical results of biochemical indicators were presented in **Figure 2A**. After 15 days of administration, the level of FBG, LDL, T-CHO, TG, Scr, BUN, β2-MG in serum, and UP in urine of DN rats increased significantly compared with that of control rats (*P* < 0.05 or *P* < 0.01 or *P* < 0.001). After treatment of HK (0.75 g/kg⋅d), YX (27 mg/kg⋅d), DHYL (4.3 g/kg⋅d), DHYH (7.2 g/kg⋅d), JNL (216 mg/kg⋅d), JNH (360 mg/kg⋅d), the levels of these indicators were nearly restored to normal. The results of the histological examination were consistent with the biochemical analysis (see **Figure 2B**). Compared with the control group, the DN model group showed renal tubular epithelial vacuolar degeneration, interstitial inflammatory cell infiltration, glomerular and tubular atrophy and interstitial fibrosis. Western blotting analysis (**Figure 2C**) showed the expression of Wnt4, β-catenin, TGF-β1 in renal tissue of control rats were significant from that in DN rats (*P* < 0.05). Compared with DN model group, the levels of creatinine, urea nitrogen, 24-h urinary protein and renal histopathology were significantly improved in the administration groups, and the expression levels of Wnt4, β-catenin and TGF-β1 protein was significantly decreased, especially TLR treatment.

### Effect of TLR and DTG on GMCs Hypertrophy

**FIGURE 3:**
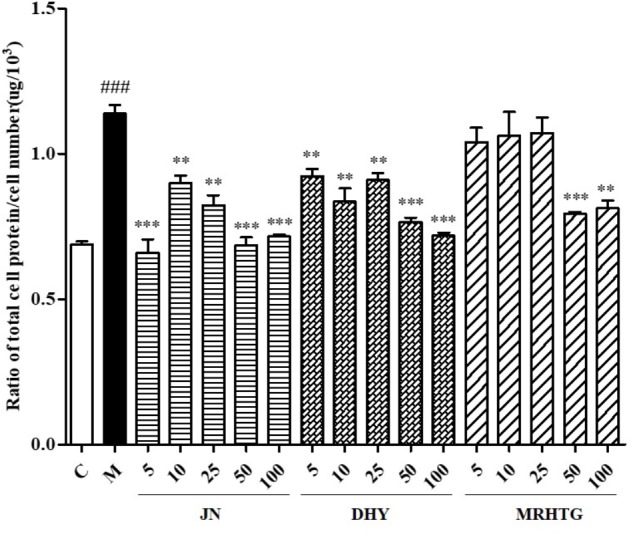
Effects of DHY, JN, and MRHTG on hypertrophy of high glucose-induced GMCs. ^###^*P* < 0.001: model group vs. control group; ^∗∗^*P* < 0.01, ^∗∗∗^*P* < 0.001: after treatment by DHY, JN, and MRHTG vs. model group. Data were presented as the mean ± SE.

As shown in **Figure 3**, high glucose increased the ratio of total protein to total cells in GMCs, resulting in cell hypertrophy, whereas TLR, DTG and the major active ingredient acteoside (5, 10, 25, 50, and 100 μmol⋅L^-1^), the ratio of total protein to total cell number decreased to a certain extent in a dose-dependent manner.

### TGF-β1 and Wnt/β-Catenin Signaling Pathway on GMCs Treated With TLR and DTG

**FIGURE 4:**
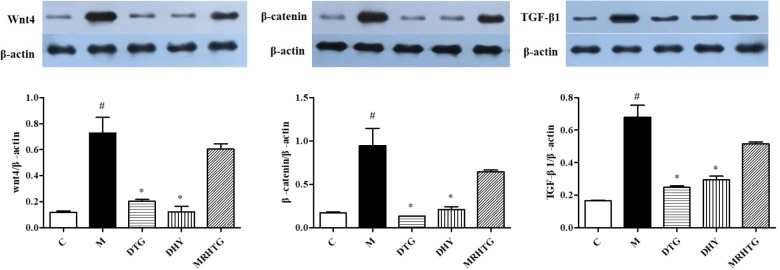
Effects of DHY and JN on the expression of Wnt-4, β-catenin and TGF-β1 in GMCs. ^#^*P* < 0.05: model group vs. control group; ^∗^*P* < 0.05: after treatment by DHY, JN, and MRHTG vs. model group. Data were presented as the mean ± SE.

As shown in **Figure 4**, the protein expression levels of Wnt4, β-catenin and TGF-β1 were significantly increased in high glucose-induced GMCs (*P* < 0.05). The expression of Wnt4, β-catenin and TGF-β1 returned to the normal level after intervention of TLR and DTG, but there was no significant effect of the MRHTG group on the expression levels of Wnt4, β-catenin and TGF-β1.

### Metabolomics Insights

#### QC samples Analysis

**FIGURE 5:**
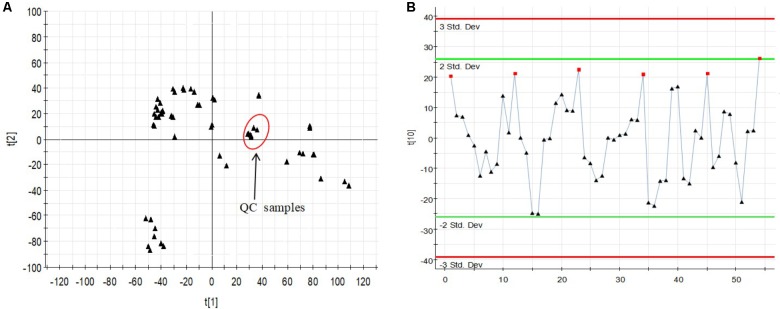
Assessment of QC samples **(A)** PCA score plot (PC1 vs. PC2) of test samples and QC samples. **(B)** Trend plot showing the variation of *t*[1] over all observations. QC samples were colored as red boxes and test samples were colored as black triangle. *X*-axis numbers represented sample number (54 injections). *Y*-axis was arbitrary (3 SD).

The relatively clustering of QC samples (**Figure 5A**) and relative standard deviations (RSD%) of ion intensity (**Table 1**) expound and prove the quality of QC data. The trend plot showing the variation of *t*[1] over all observations (**Figure 5B**). The extracted ion chromatographic peaks of 10 ions were selected for method validation. The repeatability of method was evaluated by using six replicates of QC sample. This type of results demonstrated that the method had excellent repeatability and stability.

**Table 1:** Coefficient of variation of ion intensity of selected ions present in the QC samples covering the range of retention times.

*T*_R__*m/z* pairs	QC1	QC2	QC3	QC4	QC5	QC6	RSD%
1.11_136.0398	47.9838	49.0509	51.4734	49.2034	50.0331	52.3910	3.28
4.20_162.0556	95.4064	89.9217	92.6748	93.3017	95.6169	101.9378	4.29
6.27_170.0602	83.0727	85.1074	82.0747	83.6984	84.0937	73.3099	5.28
7.63_255.0649	51.3640	52.4464	48.6620	51.7397	52.9293	53.8687	3.45
10.09_149.0244	41.1500	42.0058	43.2755	41.8232	40.3977	43.2042	2.69
10.66_318.3001	48.3586	46.5870	45.4164	49.2801	44.6299	43.6165	4.73
2.84_179.0713	15.8304	16.2821	16.4652	15.2931	16.3969	15.1267	3.65
10.89_299.2012	20.8190	21.2219	21.8836	22.0706	21.5110	20.3967	2.99
4.48_146.0613	9.2522	10.0701	9.8090	8.8366	9.2831	8.9621	5.13
3.14_372.2377	26.8391	27.5472	29.8873	28.9075	26.5686	26.8156	4.85


#### Multivariate Data Analysis

**FIGURE 6:**
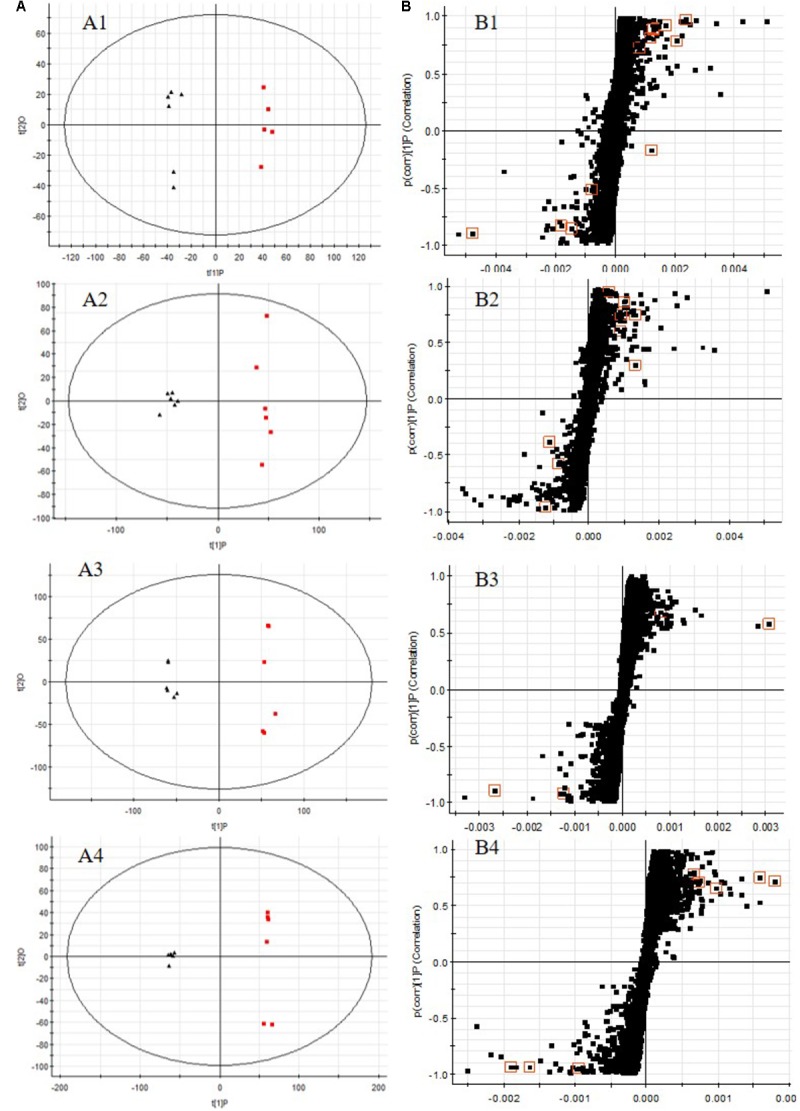
OPLS-DA scores plots **(A)**, S-plot of OPLS-DA **(B)** for serum **(A1,A2,B1,B2)** and urine **(A3,A4,B3,B4)** samples of DN model group (red) vs. control group (black) in positive **(A1,B1,A3,B3)** and negative **(A2,B2,A4,B4)** ion mode. (**A1**, *R*^2^*Y* = 0.9920, *Q*^2^ = 0.8994; **A2**, *R*^2^*Y* = 0.9882, *Q*^2^ = 0.9269; **A3**, *R*^2^*Y* = 0.9932, *Q*^2^ = 0.9363; **A4**, *R*^2^*Y* = 0.9933, *Q*^2^ = 0.9513).

All the data containing the retention time, peak intensity and exact mass were imported in the MassLynx^TM^ software for multiple statistical analyses. The supervised OPLS-DA model, a pattern recognition approach, were established to separate serum or urine samples into two blocks between DN model group and control group. The supervised OPLS-DA with 100% sensitivity and no less than 95% specificity using a leave one out algorithm showed a better discrimination between the two groups (**Figures 6A1–A4**), which demonstrated that the DN model was built successfully. Based on these results, the OPLS-DA score plot (**Figures 6B1–B4**) were used to look for potential markers associated with DN progress. The *R*^2^*Y* and *Q*^2^ of PLS-DA model in positive and negative modes for serum and urine samples were suggested that the PLS-DA model was good to fitness and prediction.

The UPLC-QTOF/MS analysis platform provided the retention time and precise molecular mass within measurement errors (<5 ppm) as well as the fragments of corresponding production for the structural identification of metabolites. According to the precise molecular mass, predicted elemental composition was predicted and potential molecular formula could be searched out in Human Metabolome Database^4^. Twenty-seven endogenous metabolites (12 in serum and 15 in urine) were tentatively identified by comparing with authentic standards or based on the protocol detailed above method. The information about the detected endogenous metabolites was summarized in **Table 2**.

**Table 2:** Potential metabolites selected and identified between DN model group and control group.

No.	*T*_R_/min	*m/z*	Metabolites	VIP^a^	Trend^b^	HMDB	Source	Pathway
Sm1	1.70	146.0605	2-Keto-glutaramic acid	5.92	↓	01552	serum	Alanine, aspartate and glutamate metabolism
Sm 2	9.71	524.3721	Guanosine triphosphate	19.10	↓	01273	serum	Purine metabolism
Sm 3	4.01	353.2471	Thromboxane A2	3.73	↑	01452	serum	Arachidonic acid metabolism
Sm 4	8.24	522.3582	LysoPC(18:1(9Z))	14.38	↑	02815	serum	Glycerophospholipid metabolism
Sm 5	9.62	482.3251	Ceramide (d18:1/12:0)	4.42	↑	04947	serum	Sphingolipid metabolism
Sm 6	3.40	498.2887	Taurochenodesoxycholic acid	4.87	↓	00951	serum	Primary bile acid biosynthesis
Sm 7	6.15	378.2404	Sphingosine 1-phosphate	3.99	↓	00277	serum	Sphingolipid metabolism
Sm 8	6.98	504.3082	Thiamine triphosphate	4.04	↓	01512	serum	Thiamine metabolism
Sm 9	8.43	464.3147	Glycocholic acid	3.27	↓	00138	serum	Primary bile acid biosynthesis
Sm 10	10.28	301.2166	Retinyl ester	4.86	↓	03598	serum	Retinol metabolism
Sm 11	11.53	442.0717	Guanosine diphosphate	3.29	↓	01201	serum	Purine metabolism
Sm 12	4.93	448.3062	Chenodeoxycholic acid glycine conjugate	3.36	↑	00637	serum	Primary bile acid biosynthesis
Um 13	3.55	245.0116	Isopentenyl pyrophosphate	3.01	↑	01347	urine	Terpenoid backbone biosynthesis
Um 14	4.51	347.1695	Inosinic acid	5.76	↑	00175	urine	Purine metabolism
Um 15	2.81	173.9946	*N*-Acetyl-L-aspartic acid	3.63	↓	00812	urine	Alanine, aspartate and glutamate metabolism
Um 16	9.84	583.3128	Cholic acid glucuronide	4.77	↑	02577	urine	Pentose and glucuronate interconversions; Starch and sucrose metabolism
Um 17	3.70	300.0536	*N*-Acetyl-D-Glucosamine 6-Phosphate	5.78	↓	01062	urine	Amino sugar and nucleotide sugar metabolism
Um 18	7.57	343.0834	Thiamine monophosphate	6.59	↓	02666	urine	Thiamine metabolism
Um 19	9.30	253.1075	Galactosylglycerol	4.64	↓	06790	urine	Galactose metabolism
Um 20	1.11	136.0398	Adenine	11.10	↓	00034	urine	Purine metabolism
Um 21	4.20	162.0557	Aminoadipic acid	16.33	↓	04077	urine	Lysine degradation
Um 22	6.26	170.0603	Cysteic acid	7.40	↓	02757	urine	Taurine and hypotaurine metabolism
Um 23	7.63	255.065	5-L-Glutamyl-taurine	3.97	↓	04195	urine	Taurine and hypotaurine metabolism
Um 24	10.66	318.3002	Phytosphingosine	3.62	↓	04610	urine	Sphingolipid metabolism
Um 25	2.83	179.0713	L-Gulonolactone	3.40	↑	03466	urine	Ascorbate and aldarate metabolism
Um 26	4.47	146.0613	2-Keto-glutaramic acid	3.37	↑	01552	urine	Alanine, aspartate and glutamate metabolism
Um 27	10.09	149.0245	2-Oxo-4-methylthiobutanoic acid	8.94	↑	01553	urine	Cysteine and methionine metabolism


*^*a*^Variable importance in the projection (VIP) values were obtained from cross-validated PLS-DA models with a threshold of 1. ^*b*^Models vs. Controls: ↑ content increased; ↓ content decreased*.

#### Changes of Relative Intensity of Endogenous Metabolites

**FIGURE 7:**
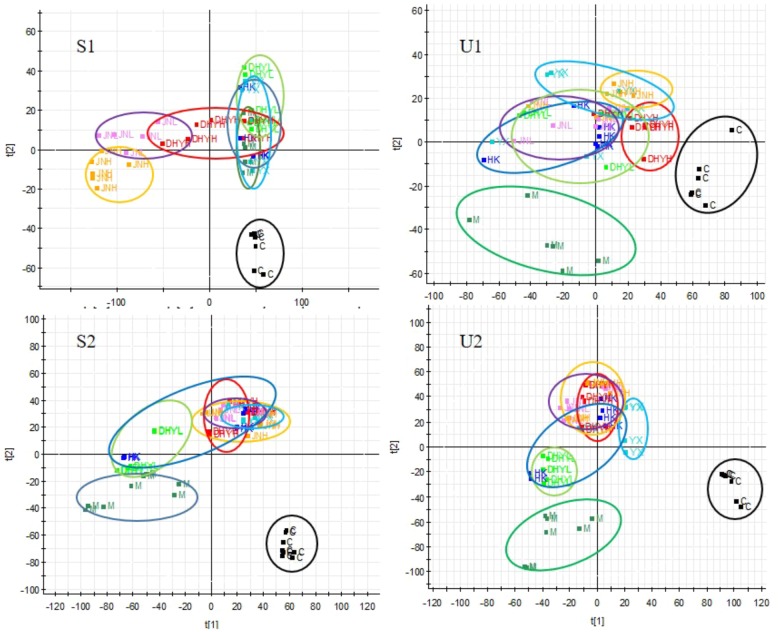
PLS-DA scores plots for serum **(S1,S2)** and urine **(U1,U2)** samples from DN model rats and controls and administration group rats in positive and negative ion mode.

**FIGURE 8:**
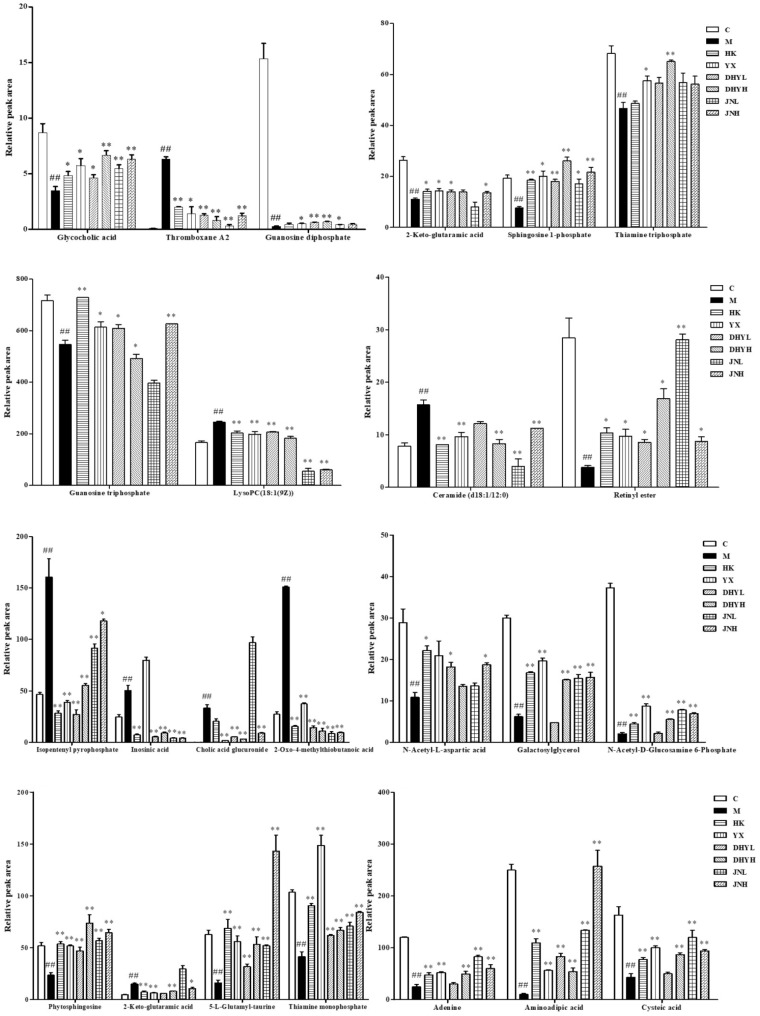
Changes in the relative intensity of target metabolites identified by UPLC-QTOF/MS. A two tailed, parametric *t*-test was used to determine the significance of the change in relative intensity for each metabolite. Bars represent the mean relative metabolite concentration and standard deviations. ^##^*P* < 0.01: model group vs. control group; ^∗^*P* < 0.05, ^∗∗^*P* < 0.01: after treatment by HK, YX, DHYL, DHYH, JNL, and JNH vs. model group. Data were presented as the mean ± SE.

In order to study the efficacy and action mechanism of TLR and DTG for treating DN disease, PLS-DA model analysis was built to obtain the changes during the control group, DN model group and administration group rats (**Figure 7**). The variations of metabolic profiling in serum and urine for administration group rats had the tendency to restore back to the levels of controls, especially TLR and DTG groups (**Table 3**). Furthermore, the relative quantities of 27 endogenous metabolites (12 in serum and 15 in urine) were ultimately identified by comparing with authentic standards or based on the protocol detailed above method. Twenty-four endogenous metabolites (except for taurochenodesoxycholic acid, chenodeoxycholic acid glycine conjugate and L-gulonolactone) in serum and urine significantly affected by TLR and DTG were restored back to a control-like level. The detailed information was showed in **Figure 8**.

**Table 3:** The relative distance between treatment groups and control group from the PLS-DA score plot (mean ± SE).

	Source	Serum		Urine	
	ESI	+	–	+	–
C	*X*-Axis	49.88	66.31	57.47	96.17
	*Y*-Axis	–50.77	–16.5	–68.63	–29.87
	M	50.51 ± 5.01	150.46 ± 5.75	126.77 ± 8.63	136.42 ± 7.81
	HK	44.61 ± 6.72	102.57 ± 10.46^∗∗^	77.52 ± 19.3^∗^	120.71 ± 7.86
	YX	35.66 ± 7.72	97.95 ± 11.71^∗∗^	44.57 ± 2.21^∗∗^	98.79 ± 6.56^∗∗^
	DHYL	28.63 ± 4.41^∗∗^	101.85 ± 9.7^∗∗^	131.48 ± 7.32	135.53 ± 0.82
	DHYH	68.71 ± 11.11	75.37 ± 2.94^∗∗^	53.11 ± 6.31^∗∗^	124.65 ± 2.76
	JNL	144.12 ± 11.05	101.95 ± 8.87^∗∗^	50.99 ± 4.71^∗∗^	128.8 ± 3.41
	JNH	177.55 ± 6.24	79.26 ± 8.64^∗∗^	51.4 ± 5.77^∗∗^	125.71 ± 2.8


*Compared with model group, ^∗^P < 0.05, ^∗∗^P < 0.01.*

#### Metabolic Pathway Analysis

**FIGURE 9:**
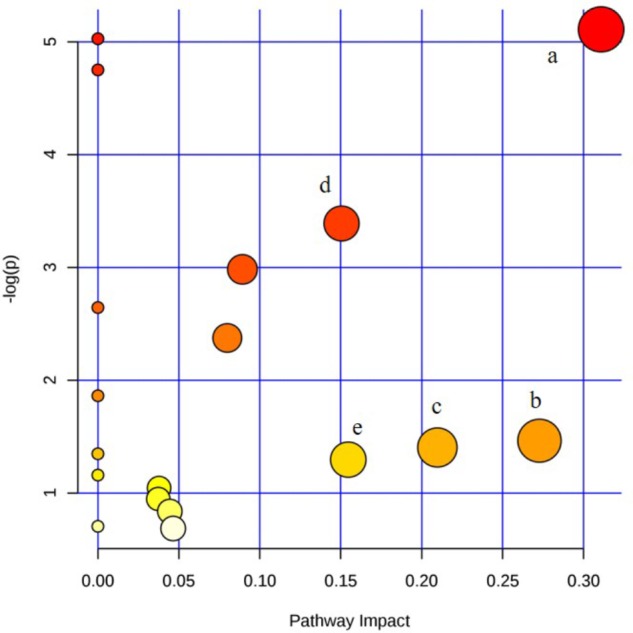
Summary of pathway analysis of serum and urine samples of rats. (a) sphingolipid metabolism; (b) pentose and glucuronate interconversions; (c) terpenoid backbone biosynthesis; (d) purine metabolism; (e) retinol metabolism.

The metabolic pathway was established by importing the potential metabolites into the web-based database MetPA. The pathway impact value calculated from pathway to topology analysis with MetPA above 0.1 was screened out as the potential target pathway. Here in **Figure 9**, for the five pathways, sphingolipid metabolism with the impact-value 0.31; pentose, glucuronate interconversion with the impact-value 0.27; terpenoid backbone biosynthesis with the impact-value 0.21; purine metabolism with the impact-value 0.15 and retinol metabolism with the impact-value 0.15 were filtered out as the most important metabolic pathways.

### Correlation Analysis

**FIGURE 10:**
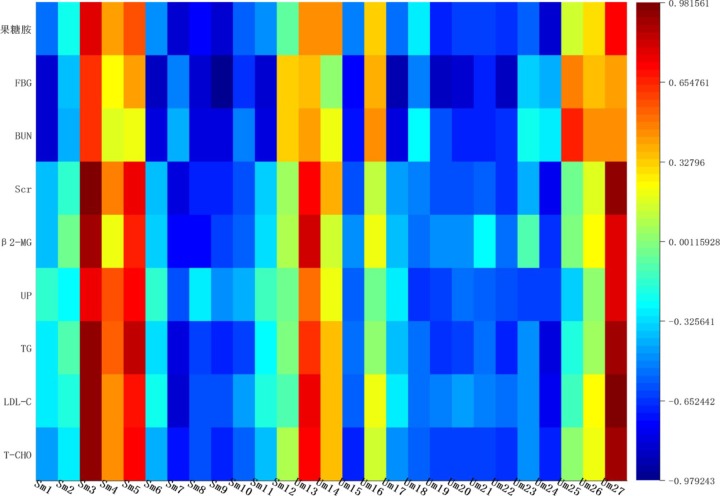
Correlation heat-map between biochemical indicators and potential biomarkers.

Correlation networks is a useful tool to elucidate the relationship between biochemical indicators and potential biomarkers, providing supports for clinical diagnosis, medical treatment and pathophysiology research. Pearson correlation coefficient *r* > 0.65 and *r* < -0.65 separately represented for significant positive correlation and negative correlation (Cai et al., 2018). Here in **Figure 10**, BUN, UP, Scr, β2-MG positively correlated with ceramide (d18:1/12:0) (*r* > 0.65); and negatively correlated with sphingosine 1-phosphate and phytosphingosine (*r* < -0.65). Ceramide (d18:1/12:0), phytosphingosine and sphingosine 1-phosphate have been found and used to explain the sphingolipid metabolism. Therefore, BUN, UP, Scr, β2-MG closely related to sphingolipid metabolism.

## Discussion

The local and traditional uses of “Dihuang” are nourishing yin and tonifying kidney. The pharmacological studies stated that “Dihuang” possesses the significant effects on cardiovascular diseases, neuroprotection, diabetes and its complications, osteoporosis, and hyperlipidemia. Moreover, acteoside, as the main active component, was reportedly possesses widely pharmacological activities, such as kidney protection, anti-oxidation, neuroprotection, liver protection, anti-cancer, and so on. But the action mechanisms still unclear completely. In our this study, the results *in vivo* showed that TLR and DTG can decrease 24-h urinary protein, serum creatine and blood urea nitrogen, alleviating the degree of renal interstitial fibrosis, reducing the expression of Wnt4, β-catenin and TGF-β1, played a role in renal protection. Moreover, the effect of the low dose group was more significant. Therefore, the optimal dose should be determined by further studies. Studies have shown that, the *in vivo* model of GMCs induced by high glucose simulates the pathophysiological changes of DN (Abboud, 2012). These *in vitro* results reveal that inhibitory effects of TLR and DTG on the hypertrophy and fibrosis of GMCs, and consistent with the *in vivo* results in this study. It is confirmed that the TLR and DTG had a better inhibiting effect on TGF-β1 and Wnt/β-catenin signaling pathway.

Notably, there are several distinct metabolic pathways in various stages of progress of DN. Ceramide (d18:1/12:0), phytosphingosine and sphingosine 1-phosphate have been found and used to explain the sphingolipid metabolism. Ceramides are one of the hydrolysis byproducts of sphingomyelin by the enzyme sphingomyelinase (sphingomyelin phosphorylcholine phosphohydrolase E.C.3.1.4.12) which has been identified in the subcellular fractions of human epidermis and many other tissues (Summers, 2006). Besides, the accumulation of ceramide can cause many lipid metabolic diseases (Li et al., 2002), and some pathogen selectively alter metabolic pathways and promote the absorption of fat into ceramides. Studies have shown that high-fat diet for 3 weeks can significantly cause ceramide and sphingomyelin in the mouse liver and nucleus accumulation, and ultimately make the liver browning and lead to fatty liver (Chocian et al., 2010). Scientists have recently demonstrated that inhibition and control of sphingolipid synthase contribute to the treatment of atherosclerosis, insulin resistance, diabetes and cardiomyopathy (Li et al., 2014). In DN, the content of ceramide increased significantly compared to normal rats and recovered to normal after administration of TLG and DTG. In recent years, studies have found that sphingosine 1-phosphate (S1P) can participate in the regulation of GMC growth and differentiation process (Spiegel and Kolesnick, 2002). Phytosphingosine is involved in diverse cell processes, including cell-cell interaction, cell proliferation, differentiation, and apoptosis. The contents of S1P and phytosphingosine decreased in DN, which was consistent with the injured of GMCs induced by DN. In addition, sphingolipid metabolic disorders may lead to cell death and are closely related to insulin-related diseases (Pang et al., 2008).

Cholic acid glucuronide has been found and used to explain the pentose and glucuronate interconversions. Cholic acid glucuronide is the glucuronidated metabolite of cholic acid, one of the four main acids produced by the liver where it is synthesized from cholesterol. Upon formation, the glucuronide is rapidly and effectively cleared from the circulation and excreted via urine (Little et al., 1985). In DN, the decreased amount of cholic acid glucuronide in urine indicated that the renal excretion was perturbed. Guanosine triphosphate, guanosine diphosphate, inosinic acid and adenine have been found and used to explain the purine metabolism. Inosinic acid is a purine nucleotide which has hypoxanthine as the base and one phosphate group esterified to the sugar moiety. The hypoxanthine in the purine metabolism oxidize to xanthine, adenosine monophosphate can degrade into uric acid and accumulate in the urine into uremic toxins, eventually leading to renal failure (Sánchez-Lozada et al., 2005; Seaman et al., 2008). Results suggested that these target pathways showed the marked perturbations over the time-course of the treatment and could contribute to the development of DN.

## Conclusion

This study showed that TLR and DTG could prevent its occurrence and development of DN by combining biochemical and pathological indicators with metabolomics technology, and by assessing comprehensive curative effect. Through the application of metabolomics technology, 27 endogenous metabolites (12 in serum and 15 in urine) could be identified in the process of DN. After drug intervention, these markers varied to some extent (*P* < 0.05). The results showed that TLR and DTG played a significant role in regulating the expression levle of TGF-β1 protein in renal tissue and mesangial cells. Therefore, to further study the mechanism of TLR on TGF-β/Smad signaling pathway, it is necessary to systematically study the protective mechanism of TLR on DN. The study paved the way for further exploration of the pathogenesis of DN, early diagnosis and the evaluation of curative effect.

## Author Contributions

SS and J-aD designed the project. XD, HC, TZ, and HY performed the experiments. ZZ, E-xS, SG, DQ, and DW provided technology guidance. XD and SS wrote and modified the manuscript.

## Conflict of Interest Statement

The authors declare that the research was conducted in the absence of any commercial or financial relationships that could be construed as a potential conflict of interest.

## Footnotes

1 http://www.metaboanalyst.ca/faces/upload/PathUploadView.xhtmlpc

2 http://www.genome.jp/kegg/

3 http://www.hmdb.ca/

4 http://www.hmdb.ca/

## Abbreviations

β2-MG: serum β2-microglobulin
BUN: serum urea nitrogen
C: control group
DHYH: TLR high dose group
DHYL: TLR low dose group
DN: diabetic nephropathy
DTG: Dihuangye total glycoside capsule
FBG: Fasting blood glucose
GMC: glomerular mesangial cells
HE: hematoxylin eosin
HK: Huangkui capsule group
JNH: DTG high dose group
JNL: DTG low dose group
LDL: low density lipoprotein
M: model group
MRHTG: Acteoside
RG: *Rehmannia glutinosa* Libosch
Scr: serum creatinine
STZ: streptozotocin
T-CHO: total cholesterol
TCM: Traditional Chinese medicine
TG: triglyceride
TLR: total glycoside extracted from leaves of *Rehmannia*
UP: urine protein
YX: Irbesartan group
